# Mystery in a Bottle: Will the EPA Require Public Disclosure of Inert Pesticide Ingredients?

**DOI:** 10.1289/ehp.118-a168

**Published:** 2010-04

**Authors:** Bob Weinhold

**Affiliations:** **Bob Weinhold**, MA, has covered environmental health issues for numerous outlets since 1996. He is a member of the Society of Environmental Journalists

For nearly two-thirds of a century, up to 99% of the ingredients in any given pesticide product have legally been hidden from public view, even though many are known to be toxic. The primary impetus has been to protect industry trade secrets. But the U.S. Environmental Protection Agency (EPA) is considering what it calls a “sea change” in policy, possibly requiring public disclosure of 100% of a pesticide product’s ingredients. Many factions have a stake in the outcome. Claudia Polsky, deputy attorney general with the California Department of Justice’s Environment Section, says the inability to publicly disclose ingredient information has left California unable to assure its residents that pesticides the state wants to use for various purposes are safe, resulting in lawsuits, injunctions, and protracted negotiations with pesticide manufacturers. Susan Smolinske, director of the Regional Poison Control Center at the DMC Children’s Hospital of Michigan, says, “We get calls where the lack of [ingredient] information results in a delay in treatment. It does cripple us.” And Aimee Code, water quality coordinator for the Northwest Coalition for Alternatives to Pesticides, says all ingredients need to be on the label because “[consumers] make choices based on that.”

But Ray McAllister, senior director of regulatory policy for the pesticide industry group CropLife America, says he’d like to keep the situation “fairly close to where it is now.” He points out that each formulated pesticide product is subject to a battery of acute toxicity tests whose results are reflected in the cautions, usage directions, and first aid statements on product labels.

However, the EPA is concerned enough about the status quo that it is investigating how to better inform consumers about most or all of the ingredients in pesticides. That could correct what the agency sees as the current “market failure” that allows pesticide products to potentially contain “levels of hazardous ingredients that are higher than society needs or wants.”

## Active Controversies

The Federal Insecticide, Fungicide and Rodenticide Act (FIFRA), a cornerstone of pesticide regulation that was implemented in 1947, set up a technical distinction between “active” and “inert” ingredients in pesticide products. Active ingredients are defined as those that are intentionally added and designed specifically to kill or control the target pest. All other ingredients, such as fragrances, dyes, aerosol propellants, solvents, desiccants, carriers, and other substances, are defined as inert.

Under FIFRA only active ingredients must be named on pesticide product labels. All inert ingredients, which can constitute more than 99% of a product, can be lumped together under the category of inert or other ingredients and listed simply as a percentage of the product’s total weight. According to the EPA, there are currently more than 1,000 active ingredients and about 4,000 inert ingredients in use.

FIFRA generally allows the identity of inerts to be kept secret to protect confidential business information. However, manufacturers must divulge all ingredients to the EPA. FIFRA gives the agency the option of requiring such ingredients to be listed on the label if they “pose a hazard to man or the environment.” Historically, however, the agency has interpreted FIFRA language primarily in favor of protecting confidential business information.

The EPA and others acknowledge the term “inert” is often popularly perceived to mean “harmless.” But as noted in 2 petitions filed in 2006 asking the EPA to disclose hazardous inerts, at least 374 such ingredients are known to present a risk of injury to human health or the environment, as determined by the EPA or other federal agencies. Among these are coal tar, dibutyl phthalate, glutaraldehyde, hexane, hydrochloric acid, kerosene, naphthalene, nitric acid, xylene, and numerous petroleum distillates and fuel oils. A further 1,863 inerts were of unknown toxicity at the time the petitions were filed. The petitions also noted that 455 substances on the EPA inerts list are also in the Hazardous Substances Data Bank, which tallies potentially toxic substances and is maintained by xvzcthe National Library of Medicine. Furthermore, 516 ingredients currently used as both active and inert ingredients (depending on the product) are listed only on those products in which they are deemed active ingredients, regardless of toxicity. The EPA says it does not have current numbers for any of these categories.

Much of the regulatory testing of pesticides focuses solely on the active ingredient. But many studies have found that a complete pesticide product can be significantly more toxic to human or environmental health than the active ingredient alone. Several examples are noted in a commentary by Caroline Cox and Michael Surgan published in the December 2006 issue of *EHP*, and studies conducted since then have continued to illustrate this phenomenon. In work published in the 19 January 2009 issue of *Chemical Research in Toxicology*, Nora Benachour and Gilles-Eric Séralini found that an inert ingredient used as a surfactant, polyethoxylated tallowamine, was more damaging than the active ingredient glyphosate to human umbilical cord, placental, and embryonic cells. Monsanto, which makes glyphosate, challenged the findings and the study methods in a press release and multiple media accounts in the months after the study was published. But the researchers maintain their methods and findings are valid and reflect possible effects at real-world concentrations.

## Movement toward Disclosure

The Government Accountability Office recognized the conflict of not listing inert ingredients, even though some are toxic, as long ago as 1975. That same year, the EPA required that a handful of known toxic inerts be identified on pesticide product labels. That number was boosted to 57 in 1987. Manufacturers quickly phased out all but 8, in part because they chose to reformulate rather than disclose such ingredients, according to the EPA. (McAllister says if more disclosure is required in the future, some companies will choose to take the same route for certain products.) Of those 8, only 5—adipic acid, bis(2-ethylhexyl) ester; ethylene glycol monoethyl ether; hydroquinone; nonylphenol; and phthalic acid, bis(2-ethylhexyl) ester—are still used as inerts.

Several thousand other inerts still remained secret, however, spurring 10 state and territorial attorneys general and scores of advocacy organizations to petition the EPA in 1998 to require complete ingredient disclosure on labels. The agency denied this request, and its decision was upheld in 2004 by a federal judge. But in light of the judge’s clarification of the FIFRA language giving the EPA authority to require disclosure of inerts known to be harmful, 15 state and territorial attorneys general (including the California Department of Justice) and 22 advocacy groups again petitioned the EPA to require disclosure in 2006. This time they focused on 374 inerts that have been designated as toxic under one federal law or another, such as the Clean Air Act, the Emergency Planning and Community Right-to-Know Act, or the Toxic Substances Control Act.

On 30 September 2009, the EPA announced it was granting the request and would pursue a change in disclosure of hazardous inerts. The agency also said it would consider more than the requested 374 ingredients for inclusion on labels due to regulatory and administrative difficulties in drawing a line on what to disclose.

## Competing Interests

The EPA doesn’t have current publicly available data on total pesticide use, although agency spokeswoman Enesta Jones says the agency expects to release a report on sales and use of pesticides later in 2010. The agency had previously calculated in *Pesticides Industry Sales and Usage: 2000 and 2001 Market Estimates* that about 5 billion pounds of active ingredients were used in 2001 in products such as insecticides, herbicides, fungicides, disinfectants, and wood preservatives. If each of the United States’ 3,717,792 square miles received the same amount, that would be an average of about 1,350 pounds per square mile per year. Based on limited data on the percentages of inert ingredients in various types of products, a conservative estimate suggests about 6–10 billion pounds of pesticide products may be spread in the environment each year.

The American Association of Poison Control Centers received 93,998 calls about pesticides in 2008, or 3.8% of all calls involving human poison exposures. Pesticides were the ninth most common topic of concern. There were an additional 3,705 calls from people asking for pesticide information; 19% were from pesticide applicators. The National Pesticide Information Center received 26,440 calls from 1 April 2008 to 31 March 2009, 88% from the general public. The center, which is jointly funded by Oregon State University and the EPA, also had 2,465,802 website hits from all over the globe. That was 1 million more than the previous year. The number of phone calls at both centers has remained fairly steady for several years.

Some doctors aren’t very concerned about acute or chronic pesticide exposures. “The chance of being injured is vanishingly small,” says Daniel Brooks, co–medical director of the Banner Poison Center in Phoenix, Arizona. He says any clinical effects are usually caused by active ingredients and that toxic exposures occur largely when pesticides aren’t used as directed on the label or when they’re intentionally ingested.

But it’s impossible to know how many people are being affected, says Catherine Karr, an executive committee member of the American Academy of Pediatrics Council on Environmental Health and director of the University of Washington Pediatric Environmental Health Specialty Unit. “We don’t have a national surveillance system for tracking pesticide exposures,” she says. “We only have some poor and limited proxies, like data gleaned from poison control center call records.”

## Protection Tradeoffs

In response to various pesticide concerns—plus the fact that products such as foods, cosmetics, drugs, and some household goods have much more transparent labeling of ingredients—the EPA issued a 23 December 2009 *Federal Register* advance notice of proposed rulemaking in which it laid out a list of inert disclosure issues, possible ways of resolving them, and a request for public comment. The agency says it is trying to help consumers and health care providers who want more information, encourage the manufacture of less-toxic products, and maintain industry competitiveness.

Marty Monell, deputy office director for management in the EPA’s Office of Pesticide Programs, says one option is labeling 100% of the ingredients. There’s a “good shot” the agency will pursue this option, she says. But a lot depends on whether disclosure will reveal critical information to competing manufacturers.

Part of that secrecy has already been breached, since some ingredients are disclosed through sources such as patents, scientific studies, reverse engineering, and Material Safety Data Sheets. McAllister says one way to help protect remaining trade secrets would be to require disclosure only of the class of the ingredient, in the same way that foods or household product labels use terms such as “natural flavor” or “fragrance.” That would prevent disclosure of certain key substances, “which is where much of the magic of a particular product comes in,” he says, noting this could work with categories such as surfactants, emulsifiers, and solvents.

Allowing inerts to be identified only by class is a concern to Smolinske, though. She cites a case in which a child was exposed to unlabeled peanut butter used as bait in an ant killer. The child, whose family has a history of peanut allergy and who had been rigorously protected from peanut exposure, is now sensitized for life and runs the risk of a severe reaction to future exposures, she says.

Even if McAllister’s group-label approach doesn’t fly, some essential trade secrets will still remain intact, says Dan Goldstein, senior science fellow at Monsanto He explains that the ingredients are just one facet of a pesticide product’s unique properties. The processes used to combine the ingredients also are important, he says, and any process more complex than simply mixing the ingredients could easily remain secret, even if a competitor tries to reverse engineer the product.

## Transforming the Rules?

Cox, who is research director for the Center for Environmental Health and one of the 2006 petitioners, says experiences with other products that require more explicit identification of ingredients has shown many pesticide product businesses likely will remain viable: “Toothpaste lists all the ingredients,” she says, “and that hasn’t stopped there being a very competitive toothpaste market.”

The EPA will consider many other issues as it makes its decision, such as how to disclose ingredients (on the label, on a website, via a telephone system, etc.), where to draw the line if less than 100% of ingredients are labeled, whether to prohibit use of any inert deemed hazardous, and how quickly to require implementation of any changes. There also could be little or no change. The agency’s direction is expected to become clearer as it develops a final rule.

For Polsky, the details matter, but the main goal is simple: “Information alone is powerful in spurring market transformation,” she says.

The public comment period, which the EPA extended by 60 days at the request of two industry representatives, ends 23 April 2010. According to EPA officials, a proposed rule could be announced by mid-2011 and possibly finalized by early to mid-2012.

## Figures and Tables

**Figure:**
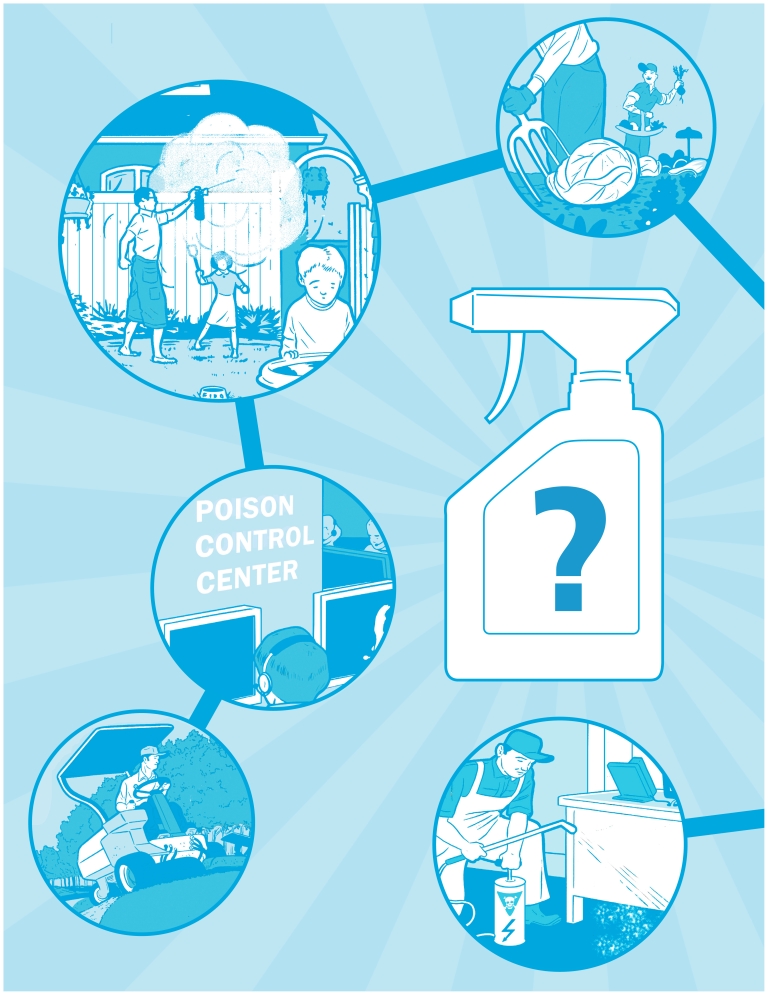


**Figure:**
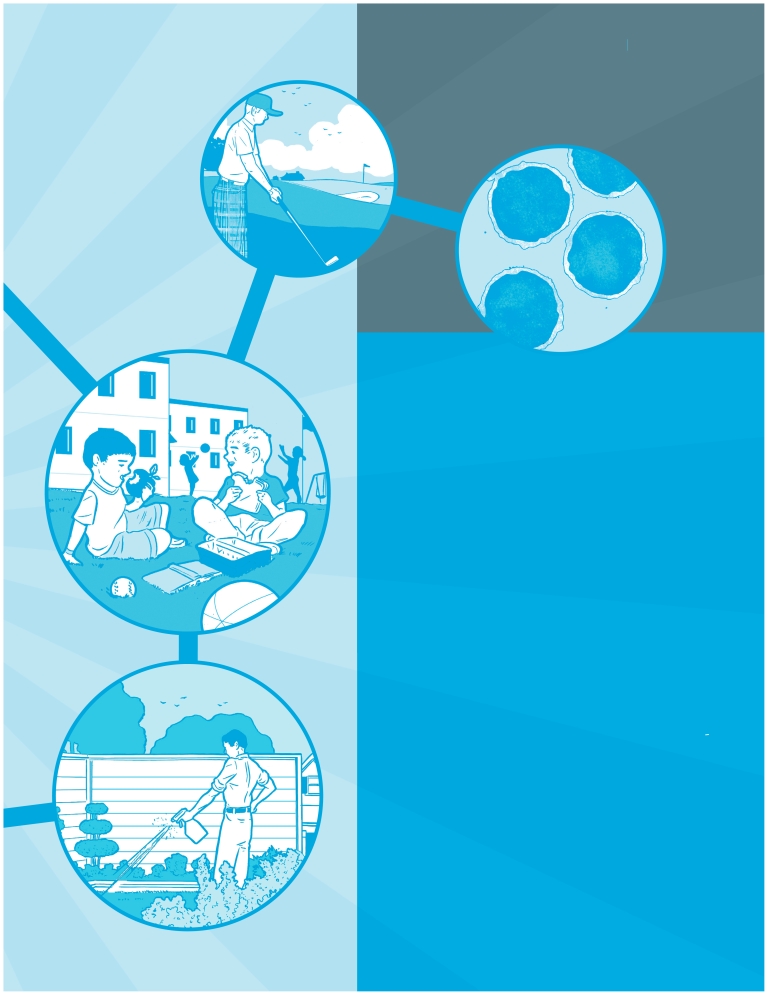
“The current lack of information [available to consumers and users] about inert ingredients interferes with the fair and efficient functioning of the market by adversely affecting consumers’ ability to exercise individual choice or express preferences and thus the market-driven incentives for producers and suppliers of pesticide products. As a result, pesticide products may contain levels of hazardous ingredients that are higher than society needs or wants and/or people may use a pesticide product or combination of products that lead to more adverse health or environmental outcomes than would otherwise occur.” —U.S. Environmental Protection Agency Advance notice of proposed rulemaking *Federal Register*, 23 December 2009

